# Comparative Mitogenomic Analyses and New Insights into the Phylogeny of Thamnocephalidae (Branchiopoda: Anostraca)

**DOI:** 10.3390/genes13101765

**Published:** 2022-09-30

**Authors:** Xiaoyan Sun, Jinhui Cheng

**Affiliations:** State Key Laboratory of Palaeobiology and Stratigraphy, Nanjing Institute of Geology and Palaeontology and Center for Excellence in Life and Palaeoenvironment, Chinese Academy of Sciences, 39 Beijing Eastroad, Nanjing 210008, China

**Keywords:** Thamnocephalidae, mitochondrial genome, phylogeny, non-monophyly of *Branchinella*

## Abstract

Thamnocephalidae, a family of Anostraca which is widely distributed on all continents of the world except Antarctica, currently consists of six genera and approximately 63 recognized species. The relationships among genera in Thamnocephalidae and the monophyly of Thamnocephalidae, determined using morphological characteristics or gene markers, remain controversial. In order to address the relationships within Thamnocephalidae, we sequenced *Branchinella kugenumaensis* mitogenomes and conducted a comparative analysis to reveal the divergence across mitogenomes of *B. kugenumaensis*. Using newly obtained mitogenomes together with available Anostracan genomic sequences, we present the most complete phylogenomic understanding of Anostraca to date. We observed high divergence across mitogenomes of *B. kugenumaensis*. Meanwhile, phylogenetic analyses based on both amino acids and nucleotides of the protein-coding genes (PCG) provide significant support for a non-monophyletic Thamnocephalidae within Anostraca, with Asian *Branchinella* more closely related to Streptocephalidae than Australian *Branchinella*. The phylogenetic relationships within Anostraca were recovered as follows: Branchinectidae + Chirocephalidae as the basal group of Anostraca and halophilic Artemiidae as a sister to the clade Thamnocephalidae + Streptocephalidae. Both Bayesian inference (BI)- and maximum likelihood (ML)-based analyses produced identical topologies.

## 1. Introduction

Order Anostraca, a group of micro-crustaceans consisting of more than 350 described species [1], live in diverse inland waters across the world [2,3,4]. The morphological architecture of Anostraca species is conservative, with a usual body length ranging from 6 to 25 mm, having 20 body segments, lacking a carapace, and bearing 11–19 pairs of leaf-like phyllopodia and stalked compound eyes [5]. Their fossil record dates back to at least the lower Cretaceous [6,7]. Anostraca are traditionally classified into two suborders: Artemiina (brine shrimp) and Anostricina (fairy shrimp) [1]. Artemiina contains two halobiont monogeneric families: Artemiidae and Parartemiidae [1,8]. Anostricina, representing nearly 86% of all Anostracan diversity, are diversified into six families: Streptocephalidae, Tanymastigidae, Branchinectidae, Thamnocephalidae, Branchipodidae, and Chirocephalidae. The monophyly of Anostraca has been supported by morphological synapomorphies [9,10,11,12,13], and confirmed by some molecular studies [14,15,16,17,18,19]. Determining the interrelationships between these eight families, however, is inherently difficult, due to the convergent morphologies of frontal and antennal appendages and abdominal outgrowths in distantly related taxa [14], furthermore, the phylogenetic relationships among some genera and families still remain unclear [14,16,20]. The family Thamnocephalidae Packard, 1883 is the most controversial and problematic regarding its phylogenetic position in Anostraca.

Thamnocephalidae currently comprises 63 valid species widespread in Europe, Asia, North and South America, southern Africa, and Australia [1]. Thamnocephalids typically have 11 pairs of thoracopods, each thoracopod with a single pre-epipodite; the vas deferens bears a dorsal loop; the male frontal appendage may be variously branched and ornamented; the male second antennal proximal antennomere can be with or without appendages; the male genitalia bears one or more longitudinal rows of spines; and the female second antennae is lamellar [5]. According to the features of the male second antennae, male genitalia, cercopods, and cephalic appendages, this family is composed of six genera and two subfamilies: *Thamnocephalus* Packard, 1877 and *Carinophallus* Rogers, 2006 of the subfamily Thamnocephalinae Packard, 1883; and *Branchinella* Sayce, 1902, *Dendrocephalus* Daday de Dées, 1908, *Phallocryptus* Birabén, 1951, and *Spiralifrons* Dixon, 2010 of the subfamily Branchinellinae Daday, 1910 [21]. Genus *Branchinella* contains two subgenera: *Branchinella* Sayce, 1902, which is endemic to Australia [8], and *Branchinellites* Daday, 1910, which is widespread in Asia and South Africa [22]. The phylogenetic position of Thamnocephalidae within Anostraca is still a subject of debate. In contemporary morphological phylogenetic schemes, the genus *Branchinella* has close affinities to *Dendrocephalus*, *Phallocryptus*, and *Spiralifrons* based mainly on the morphology of gonopods, cercopods, brood pouch, male frontal appendages, and the male distal antennomere of the second antenna. Molecular analyses, on the other hand, have indicated that Asian *Branchinella* may be closely related to the family Streptocephalidae Daday, 1910 based on 168 orthologous genes shared among 5 Anostracan species [23] and 13 Anostracan mitogenomes spanning 5~6 Anostracan genera [23,24].

Hitherto, studies on the relationships between Anostracan families and the monophyly of Thamnocephalidae have been relatively sparse, and most of phylogenetic reconstructions have been based on limited taxa sampling or individual molecular loci. The mitochondrial genome—often the workhorse for population genetic and phylogenetic studies—has been effective for recovering relationships at multiple hierarchical levels [25,26,27]. In this study, we sequence and describe the complete mitogenome of *B. kugenumaensis* samples from Suqian City, Jiangsu Province, China, which are coupled with available Anostracan mitogenomes to analyze the main features of the mitogenomes of *B. kugenumaensis* and reveal the heterogeneous sequence divergence within *B. kugenumaensis* mitogenomes. We also performed phylogenetic analyses to determine the phylogenetic relationships among *Branchinella* and assess the monophyly of Thamnocephalidae.

## 2. Materials and Methods

### 2.1. Sample Collection and DNA Extraction

We collected 42 *B. kugenumaensis* specimens from the wetlands of the Ancient Yellow River, Suqian City, Jiangsu Province, China (E118°8′, N34°1′). All samples were preserved in 95% ethanol immediately after collection. Voucher specimens (No. BKSQJS01-11) were deposited in the State Key Laboratory of Palaeobiology and Stratigraphy, Nanjing Institute of Geology and Palaeontology, Chinese Academy of Sciences, Nanjing, China. Total genomic DNA was extracted from the stored specimens using a DNeasy tissue kit (Qiagen, Hilden, Germany), following the manufacturer’s instructions.

### 2.2. PCR Amplification, Sequencing, Sequence Assembly, and Gene Annotation

Amplification and sequencing were performed according to the methods previously described by Sun [28], and assembly of mtDNA fragments, mitogenome annotation, and comparison were conducted following Sun and Cheng [29]. The sixteen pairs of PCR primers used in the present study are provided in Supplementary Table S1.

### 2.3. Analysis of Sequence Divergence

The total number of single-nucleotide polymorphisms (SNP) and indel sites were determined using the DnaSP 6.10 software [30] and manually checked. The nucleotide diversity (π) was determined using DAMBE 6 [31]. The numbers of synonymous (Ks) and non-synonymous (Ka) substitutions for each species pair were estimated using the PAML package 4.7 [32].

### 2.4. Phylogenetic Analysis

In total, mitochondrial *16S rRNA* genes of 41 specimens and mitochondrial cytochrome C oxidase subunit I (*cox1*) genes of 30 specimens were retrieved from GenBank, representing 38 species, 12 genera, and 6 families of Anostraca (Table S2). Three species belonging to the Branchinectidae clade and five species belonging to the Chirocephalidae clade were used as outgroups. We estimated the taxonomic status within genus *Branchinella* by reconstructing a phylogenetic tree using *16S rRNA* datasets. To better resolve the internal phylogeny of Thamnocephalidae, we then reconstructed the phylogenetic tree using *16S* + *cox1* datasets. In order to confirm the relationships between Thamnocephalidae and the related families, we conducted phylogenetic analyses with 27 complete mitogenomes of Branchiopoda, representing 16 species, 7 genera, and 5 families of Anostraca (Table S3). Three Spinicaudatan species and six Notostracan species were used as outgroups. The amino acid sequences of 13 PCGs were aligned using MUSCLE implemented in the MEGA X software [33]. The corresponding nucleotide sequences for each PCG were aligned using the aligned amino acid sequences implemented in DAMBE 6 [31]. Ribosomal RNAs were aligned by published rRNAs secondary structures for *Triops granarius* (Lucas, 1864) [29] in order to improve both the alignment and the tree reconstruction processes of rRNA data sets. We estimated saturation for four subset types of the concatenated data set: the first codon positions (nt1), second codon positions (nt2), the first codon positions + second codon positions (nt12), and the third codon positions (nt3) of PCGs. The best-fit nucleotide substitution models were determined by jModelTest version 0.1.1 software [34]. ProtTest 3 [35] was used for the amino acid dataset. The best selected partition schemes and models are listed in Table S4. Phylogenetic reconstructions were conducted using ML and BI methods. ML was performed with RAxML 7.0.3 [36], with the most appropriate substitution model for each of the separate partition (1000 bootstrap). BI was performed with MrBayes 3.2 [37], with two simultaneous runs (4 chains) for 10 million generations, sampled every 100 generations, of which the first 25% were discarded as burn-in. Convergence was assessed using the Tracer v1.5 software [38].

## 3. Results and Discussion

### 3.1. General Features of B. kugenumaensis Mitogenomes

**Figure 1:**
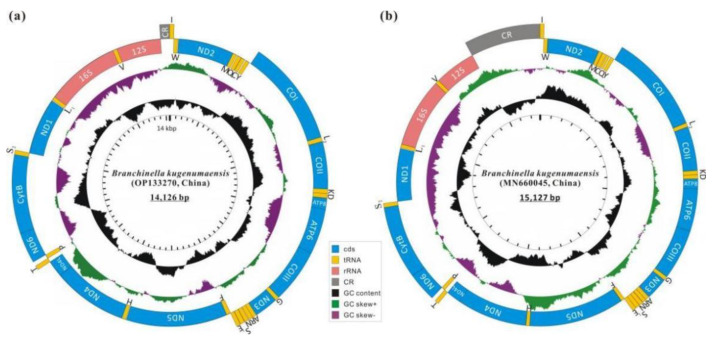
Structural representations of the mitochondrial genomes of *B. kugenumaensis* from Suqian (**a**) and Jinhua (**b**). Circular maps were drawn with CGView [40] and then modified manually. Genes shown at the outer circle are encoded by the J strand (Foward), and the genes shown at the inner circle are encoded by the N strand (Reverse). The blue, orange, pink, and grey blocks indicate PCGs, tRNAs, rRNAs, and control region, respectively. The tRNAs abbreviations: L1 = *trnL*(CUN); L2 = *trnL*(UUR); S1 = *trnS*(AGN); S2 = *trnS*(UCN).

The complete mitochondrial genome for *B*. *kugenumaensis* from Suqian City, Jiangsu Province of China, was sequenced, assembled, and deposited in GenBank (accession number: OP133270). The total length of the mitogenome was 14,126 base pairs (bp), consistent with the previously sequenced mitogenome of *B*. *kugenumaensis* from Naruto City, Tokushima Prefecture, Japan (MW136376) [28]: 14,123 bp; (Table 1, Figure 1). The complete mitogenome of *B*. *kugenumaensis* from Jinhua City, Zhejiang Province, China (MN660045) [39], had longer total length (15,127 bp) due to an expanded control region (1182 bp). Similarly, the gene arrangement in the mitogenomes of *B*. *kugenumaensis* from the 3 localities consisted of 13 PCGs, 22 tRNAs, two mitochondrial rRNAs (*rrnS* and *rrnL*), a putative control region, and a large number of intergenic sequences. Twenty genes were encoded by the majority strand (J-strand) and seventeen by the minority strand (N-strand). The canonical “ATN” start codon was the most commonly used start codon in the core PCGs of *B*. *kugenumaensis*, with the exception of the *cox1* and NADH dehydrogenase, subunit 1 (*nad1*) genes in *B*. *kugenumaensis* of Naruto and Suqian (MW136376 and OP133270), which used TTG as start codons. In *cox1*, *nad1*, NADH dehydrogenase, subunit 5 (*nad5*), and ATP synthase subunits 6 (*atp6*) genes of *B*. *kugenumaensis* from Jinhua (MN660045), TTG and GTG were used as start codons (Table 1). TAA/TAG was most commonly used as a stop codon in core PCGs of *B*. *kugenumaensis* mitogenomes, except for *cox1*, cytochrome C oxidase subunits III (*cox3*), and NADH dehydrogenase; subunit 4 (*nad4*) in *B*. *kugenumaensis* from Naruto and Suqian, and *cox1*; and cytochrome C oxidase subunits II (*cox2*), *cox3*, *nad4* and *nad5* in *B*. *kugenumaensis* from Jinhua, which ended with truncated termination codons (T), see Table 1.

The A + T content and skewness levels for the major strands of *B. kugenumaensis* and *Streptocephalus cafer* (Lovén, 1847; sensu [41]) are summarized in Table S5. All were AT-rich (67.78~68.17%) throughout the entire genomes of both species. Little heterogeneity was observed for the AT%, AT- and GC-skews in Naruto and Suqian samples. The A + T content for PCGs was the lowest in *cox1* (62.88% for Jinhua sample and 63.46% for Suqian sample) and highest in NADH dehydrogenase, subunit 6 (*nad6*) gene (76.29% for Suqian sample and 73.6% for Jinhua sample). Among the three codon positions within the 13 PCGs, the highest AT% was found for the third codon position, which was in accordance with some pancrustacean groups (see, e.g., [42]). Positive AT skew values were observed only for the entire mitogenomes and tRNA, confirming the existence of more adenine than thymine in this organism. For GC-skew, the first codon position of PCGs, rRNA, tRNAs, *cox3*, and *nad1* were markedly positive, whereas the other part of mitogenomes showed negative values.

The patterns of amino acid composition and synonymous codon usage were similar in the three mitogenomes of *B*. *kugenumaensis* (Figure S1a). The relative synonymous codon usage (RSCU) analysis revealed that all codons were present in the PCGs, except for AGG being absent in Jinhua and Suqian sequences. The five most frequently used codons (RSCU > 2) in *B*. *kugenumaensis* from Naruto and Suqian(MW136376 and OP133270) were Ser2 (UCU), Leu1 (CUU), Pro (CCU), Thr (ACU), and Ala (GCU), while *B*. *kugenumaensis* from Jinhua (MN660045) had Ser2 (UCA) instead of Thr (ACU); see Figure S1b.

### 3.2. Sequence Divergence within B. kugenumaensis Mitogenomes

The two mitogenomes of *B*. *kugenumaensis* from Naruto and Suqian (MW136376 and OP133270) were similar with 1% nucleotide dissimilarity (141 SNPs) across the complete mitogenome alignment, with 106 SNPs distributed across the 13 PCGs, of which NADH dehydrogenase, subunit 4L (*nad4L*) gene (264 bp) comprised the highest proportion (1.89%) relative to the size of the gene, whereas NADH dehydrogenase, subunit 3 (*nad3*) gene (345 bp) had the lowest proportion (0.29%). Notably, a high level of variation was detected in *B*. *kugenumaensis* from Jinhua. It was unexpected that a total of 2322 SNPs distinguished *B*. *kugenumaensis* from Jinhua and Suqian in the PCGs. There were 255 SNPs (11%) detected in the *cox1* gene, which has been proposed as the universal barcode locus for Anostraca. High-sequence variation was also observed in the tRNA genes, excluding tRNA-*Met*, tRNA-*Glu*, and tRNA-*Ser1*, which were perfectly conserved (Figure S2). A total of 115 SNPs occurred in the 22 mtDNA-encoded tRNA genes between *B*. *kugenumaensis* from Jinhua and Suqian (MN660045 and OP133270) and 45 SNPs in the stems. Instead, only three tRNA SNPs were identified in *B*. *kugenumaensis* from Suqian and Naruto (OP133270 and MW136376).

**Figure 2:**
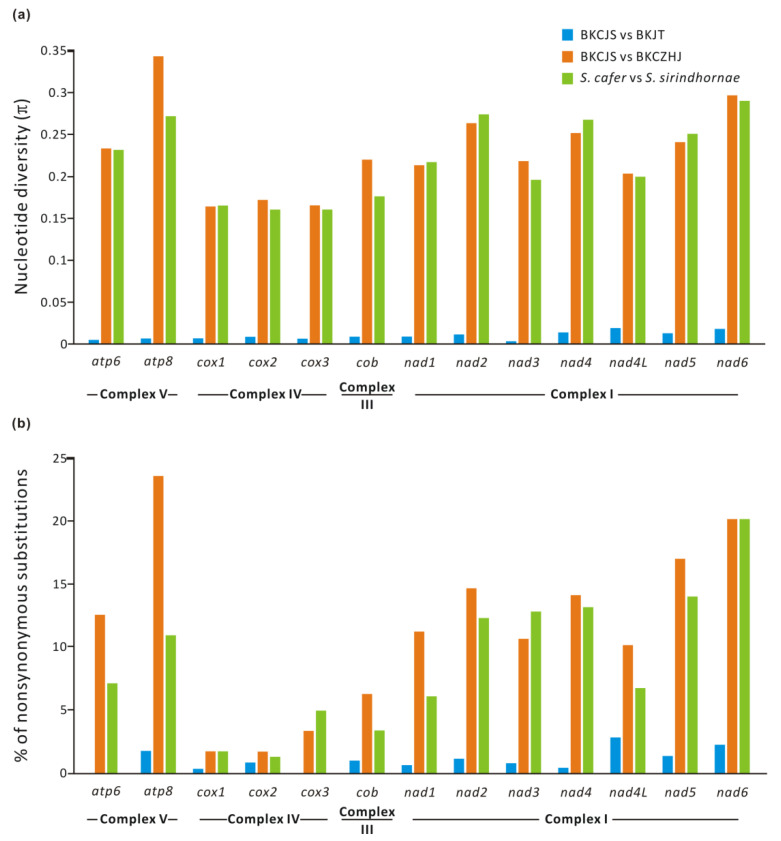
Sequence divergence across mitochondrial PCGs in *B*. *kugenumaensis* and interspecific variation in *Streptocephalus*. (**a**). Nucleotide diversity. The nucleotide diversity is plotted on the *y*-axis, and the PCGs are plotted on the *x*-axis. (**b**). Proportion in amino acid change per PCG. Abbreviations: BKCJS = *B. kugenumaensis* (Suqian, Jiangsu Province, China); BKJT = *B. kugenumaensis* (Naruto, Tokushima Prefecture, Japan); BKCZHJ = *B. kugenumaensis* (Jinhua, Zhejiang Province, China); *S.* = *Streptocephalus*.

The overall level of mitogenome diversity, represented by the mean pairwise differences per site (π), between the *B. kugenumaensis* mitogenome sequences OP133270 and MN660045 was 22.0% for the 13 PCGs concatenated, which is much higher than that of observed between the *B. kugenumaensis* mitogenome sequences OP133270 and MW136376 (π = 0.99% for 13 PCGs). The high value was on the same order of magnitude as for species of *Streptocephalus* (π = 21.5%). For each of the 13 PCGs, the diversity of nucleotides ranged from 0.029% (*nad3*) to 1.92% (*nad4L*) between OP133270 and MW136376. Nevertheless, the average values of nucleotide diversity for *B*. *kugenumaensis* from Jinhua (MW136376) was much higher, ranging from 16.4% (*cox1*) to 29.64% (*nad6*), comparable to interspecific variation for species of *Streptocephalus* (27.46% ≥ π ≥ 16.%; Figure 2a).

The proportion of amino acid change for each of the PCGs ranged from 0% (*cox3* and *atp6*) to 2.88% (*nad4L*) in the two *B*. *kugenumaensis* mitogenome sequences MW136376 and OP133270 (Figure 2b; Table S6). Meanwhile, more divergence was observed in *B. kugenumaensis* mitogenome sequence MN660045: 10.1–23.64% for complex I genes and ATP synthase (complex V) genes and 1.75–3.41% for complex IV genes (Table S6), which was also comparable to interspecific comparison for species of *Streptocephalus* (Figure 2b).

Comparing the rate of non-synonymous (Ka) and synonymous nucleotide substitutions (Ks) is important in understanding the dynamics of molecular sequence evolution [43,44,45,46]. Here, among the 13 genes of the *B*. *kugenumaensis* mitogenome sequences OP133270 and MW136376, ATPase synthase 8 (*atp8*) and *nad4L* genes had a Ka/Ks value greater than 1, suggesting that they were under positive selection during evolution and remained fixed in the population. Interspecific comparison for species of *Streptocephalus* presented an average Ka/Ks ratio ranging from 0.0063 in *cox2* to 0.26 in *atp8*, and *B*. *kugenumaensis* from Jinhua (MN660045) had an average Ka/Ks ratio ranging from 0.0067 in *cox2* to 0.25 in *atp8*, signifying lesser amino acid changes and indicating purifying selection.

### 3.3. Phylogenetic Analyses

#### 3.3.1. Phylogenetic Position of *B. kugenumaensis* from Jinhua, Zhejiang Province (MN660045)

**Figure 3:**
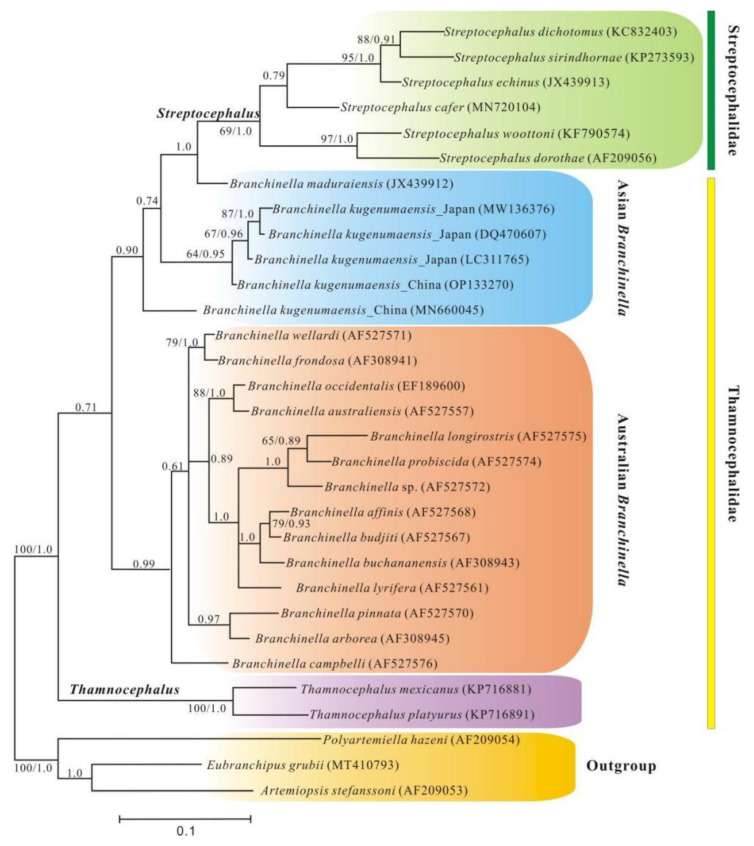
Phylogenetic trees showing the branching pattern within Thamnocephalidae (colour-coded) based on BI and ML of *16S rRNA* gene. Bootstrap support values/Bayesian posterior probabilities are shown at the nodes. Support values less than 0.60 (BI) and 60 (ML) are not shown.

Phylogenetic analyses using *16S rRNA* sequence indicated that *Branchinella* was initially divided into three major clades: Australian *Branchinella*, *B. maduraiensis,* and other Asian *Branchinella* (Figure 3). In the clade of Australian *Branchinella*, our phylogenetic reconstruction was largely congruent with the results of previous studies [47,48]. However, our phylogenetic analysis placed *B. campbelli* at a basal position within Australian *Branchinella*, which was the major difference from those results. Interestingly, *B. maduraiensis* was closely related to *Streptocephalus* (Bayesian posterior probability, BPP = 1.0), suggesting that *Branchinella* is not a monophyletic group. *B*. *kugenumaensis* from Suqian was sister to *B*. *kugenumaensis* from Naruto, whereas *B*. *kugenumaensis* from Jinhua formed a separate branch from all the other Asian *Branchinella* and was basal within this clade (BPP = 0.90). Both the ML and BI trees were highly similar in topology (Figure 3). The sequence identity for each PCG in the pairwise mitochondrial genomes of *B. kugenumaensis* also indicated that sequences from Naruto had around 16.46–29.78% divergence from Jinhua sequences and 0.3–1.89% from the Suqian sequences (Table S5). The results of the phylogenetic and genetic analyses support the idea that *B. kugenumaensis* from Jinhua is a different species. This study is the first to include three species of subgenus *Branchinellites.* Whether *Branchinellites* is monophyletic remains an open question, and more molecular loci and taxa are needed in future surveys to improve the resolution within *Branchinella*.

#### 3.3.2. Paraphyly of Thamnocephalidae

**Figure 4:**
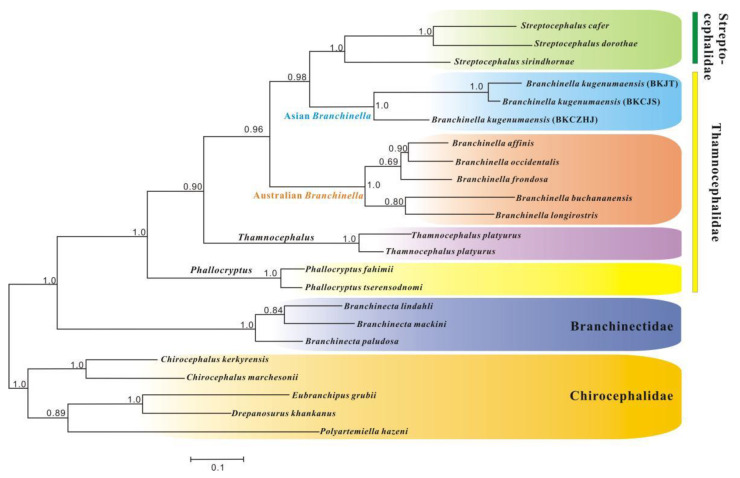
Bayesian tree of Thamnocephalidae (color-coded) derived from mitochondrial *16S rRNA* and *cox1* gene sequence datasets. Bayesian posterior probability support values are reported on the nodes.

The monophyly of Thamnocephalidae has been questioned using different molecular datasets [23,24]. Increased sampling across thamnocephalid mtDNAs has provided valuable information for understanding the phylogeny of Thamnocephalidae. The reconstruction of the phylogenetic relationships within Thamnocephalidae based on *16S* + *cox1* is presented in Figure 4. Contrary to traditional morphological taxonomy, the BI tree indicated that *Thamnocephalus* was sister to the clade *Branchinella* + *Streptocephalus* (BPP = 0.90), while *Phallocryptus* constituted the sister group of all other clades in Thamnocephalidae (BPP = 1.0). The non-monophyly of Thamnocephalidae was revealed with good support in this study, which was congruent with previous studies [23,24]. Furthermore, we identified that Asian *Branchinella* clustered to *Streptocephalus* (Streptocephalidae) as a sister group (BPP = 0.98), rather than Australian *Branchinella*. Although we clarified some relationships in *Branchinella*, it must be taken with caution, as only species of the genera *Thamnocephalus*, *Phallocryptus*, and *Branchinella* have been investigated so far. Future research is still needed with increased species sampling from the genera of *Carinophallus*, *Dendrocephalus,* and *Spiralifrons* in order to explore the detailed relationships among genera of Thamnocephalidae and Streptocephalidae.

#### 3.3.3. Phylogenetic Relationships among Anostracan Families

**Figure 5:**
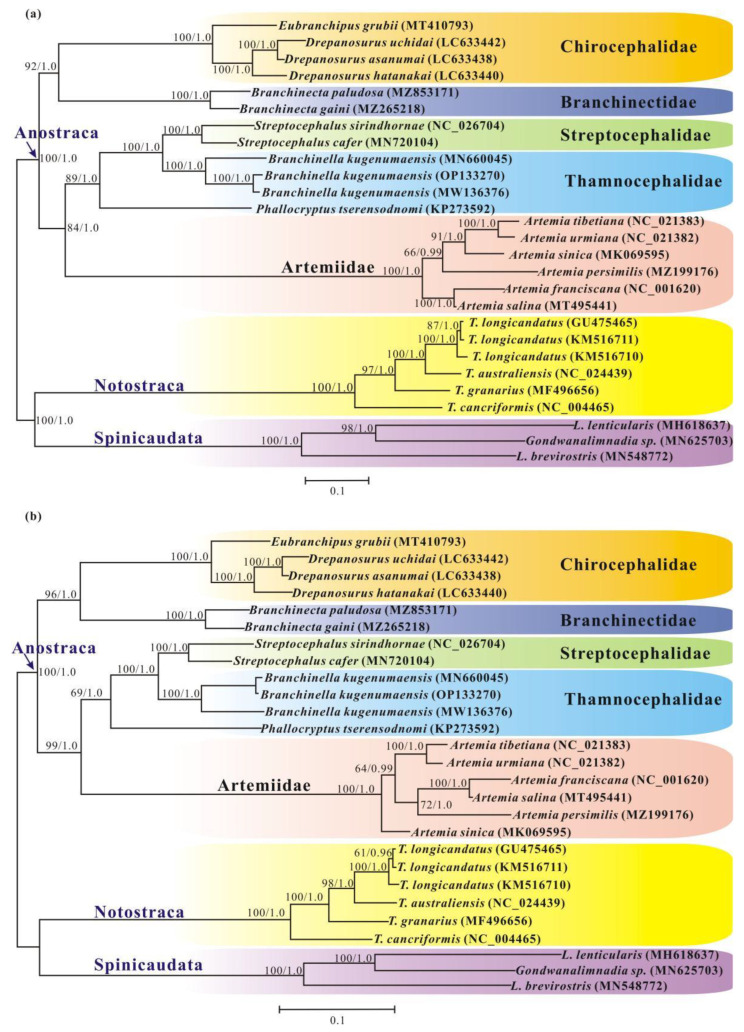
Phylogenetic trees showing relationships among Anostraca based on BI and ML of the mitochondrial nucleotide (**a**) and amino acid datasets (**b**). Bootstrap support values/Bayesian posterior probabilities are shown at the nodes.

With respect to 11 additional branchiopod mitogenomes (Tables S3 and S4), both BI and ML analyses based on nucleotide sequence data from 9 PCGs of the 16 Anostracan mitogenomes produced identical topologies with similar branch lengths, and most clades had high ML bootstrap support or BI posterior probabilities (Figure 5a). Both the BI and ML trees strongly supported the monophyly of the order Anostraca (Bootstrap support value, BP = 100, BPP = 1.0). Meanwhile, the phylogenetic topology of monophyletic *Streptocephalus* nested within a paraphyletic Thamnocephalidae was confirmed (BP = 100, BPP = 1.0).

The monophyly of Anostraca and the major clade assignments of Anostraca (halophilic Artemiidae + *Paratemia* separate from the other Anostracan families) have been broadly accepted according to molecular-based phylogenies [14,17,20,24]. However, this traditional classification of Anostraca has been challenged by molecular analysis carried out on large-genome-scale molecular sequences, supporting Branchinectidae as the basal clade of Anostraca [23]. In this study, Anostraca was clearly separated into two major groups—(1) Branchinectidae, together with Chirocephalidae, as the basal group of Anostraca; and (2) halophilic Artemiidae as a sister to the clade Thamnocephalidae + Streptocephalidae—with high support values (BP = 84, BPP = 1.0). Such a relationship has also been supported previously by a morphological study [9] and molecular analysis based on large-genome-scale molecular sequencing [23]. In addition, when they were applied to amino acid sequences of nine PCGs, the BI and ML methods supported almost identical phylogenetic relationships, except for the relationships within Artemiidae (Figure 5b).

The present phylogenetic analyses shed new light on the evolution of morphological traits. Our phylogenetic analysis confirmed the paraphyletic status of Thamnocephalidae, suggested that the diagnostic characteristics seem to be plesiomorphic (e.g., eversible portion becoming explanate distally; second maxillae with single apical setae). One or more longitudinal rows of spines on the genitalia and females with an elongated brood pouch, extending to the base of the fourth to seventh abdominal segments, may be ancestral characteristics of Thamnocephalidae+ Streptocephalidae. The present phylogenetic analyses support that Branchinectidae + Chirocephalidae were the first to diverge from the Anostraca, which implies that each thoracopod bearing a single pre-epipodite and distinct seminal vesicle were already present in the ground pattern of Anostraca. Furthermore, the origin of the halophilic Anostracans is more complex than expected and needs further investigation.

## Figures and Tables

**Table 1:** Annotation of genes in three *B. kugenumaensis* mitogenomes from different locations.

Gene	Strand ^a^	OP133270 and MW136376			MN660045		
		Size (nts)	Initiator/Terminator	IGN ^b^	Size (nts)	Initiator/Terminator	IGN ^b^
*trnI*	+	64		3	64		1
*trnW*	-	63		5	65		3
*nad2*	-	888	ATA/TAA	0	888	ATA/TAA	0
*trnM*	-	63		18, 16	63		3
*trnQ*	-	67		4	68		6
*trnC*	-	61		24, 10	60		20
*trnY*	-	62		23, 28	62		15
*cox1*	+	1534	TTG/T	0, −4	1534	TTG/T	0
*trnL2*-UUR	+	63, 64		0, 3	64		0
*cox2*	+	687	ATG/TAA	−5, 0	682	ATG/T	0
*trnK*	+	65		5, 2	65		0
*trnD*	+	64		0, 4	62		0
*atp8*	+	159	ATT/TAA	−7	159	ATT/TAA	−7
*atp6*	+	660	ATG/TAA	−1	660	GTG/TAA	−1
*cox3*	+	784	ATG/T	0, −1	784	ATG/T	−2
*trnG*	+	61		0, 1	61		0
*nad3*	+	345	ATT/TAG	−2	345	ATT/TAA	5
*trnA*	+	63		0	62		0
*trnR*	+	61		7, 8	61		2
*trnN*	+	65, 63		0, 1	65		0
*trnS1*-AGN	+	67, 65		0, 1	67		−1
*trnE*	+	63		−2	64		−3
*trnF*	-	63		−1, 0	63		0
*nad5*	-	1614	ATT/TAA	12	1624	TTG/T	0
*trnH*	-	61		0	63		0
*nad4*	-	1195	ATG/T	−8	1195	ATG/T	−8
*nad4L*	-	264	ATT/TAA	1	258	ATT/TAA	4
*trnT*	+	64		0	63		0
*trnP*	-	61		2	61		2
*nad6*	+	450	ATT/TAA	−1	450	ATT/TAA	−1
*cytb*	+	1137	ATG/TAA	−2	1137	ATG/TAA	−2
*trnS2*-UCN	+	66		−2	66		−2
*nad1*	-	897	TTG/TAA	11	897	TTG/TAA	11
*trnL1*-CUN	-	62		0	62		0
*rrnL*	-	1181, 1179		0	1180		0
*trnV*	-	65		0	65		0
*rrnS*	-	707, 706		0	709		0
CR	+	147		0	1182		0

^a^ Plus strand(+)/minus strand (-); ^b^ Number of intergenic nucleotides.

## Data Availability

All gene sequence data are available from GenBank (http://www.ncbi.nlm.nih.gov, accessed on 25 August 2022).

## Supplementary Materials

The following supporting information can be downloaded at: https://www.mdpi.com/article/10.3390/genes13101765/s1, Figure S1: Amino acid composition (a) and relative synonymous codon usage (b) in the mitogenomes of *B*. *kugenumaensis*; Figure S2: Polymorphisms in inferred secondary structures of 22 transfer RNAs found in the mitogenomes of *B*. *kugenumaensis*; Table S1: List of primer combinations used to amplify the mitochondrial genome of *B**. kugenumaensis**;* Table S2: COI & 16S rRNA sequences of Anostraca retrieved from GenBank used in this study; Table S3: Details of species and mitogenomes of Branchiopoda used in this study; Table S4: Partition schemes and best-fitting models for phylogenetic analyses; Table S5: A + T content (%) and skewness levels calculated for major strand of *B**. kugenumaensis* and *S**. cafer**;* Table S6: Nucleotide identity (%) and number of codon substitutions for each PCG of three *B. kugenumaensis* mtgenomes. The references [49,50,51,52,53,54,55,56,57,58,59,60,61,62,63,64] are cited in the Supplementary Materials.
