# Supplementary material for: Comparative Mitogenomic Analyses and New Insights into the Phylogeny of Thamnocephalidae (Branchiopoda: Anostraca)
Source: Genes (Basel). 2022 Sep 30;13(10):1765. doi: 10.3390/genes13101765 (PMC9602129; doi:10.3390/genes13101765)
Supplement: Supplementary file 1 [file genes-13-01765-s001.zip › genes-1915466-supplementary.pdf]

genes

MDPI

Supplementary Materials

Comparative Mitogenomic Analyses and New Insights into the Phylogeny of Thamnocephalidae (Branchiopoda: Anostraca)

Xiaoyan Sun and Jinhui Cheng *

State Key Laboratory of Palaeobiology and Stratigraphy, Nanjing Institute of Geology and Palaeontology and Center for Excellence in Life and Palaeoenvironment, Chinese Academy of Sciences, 39 Beijing Eastroad, Nanjing 210008,China

* Correspondence: jhcheng@nigpas.ac.cn

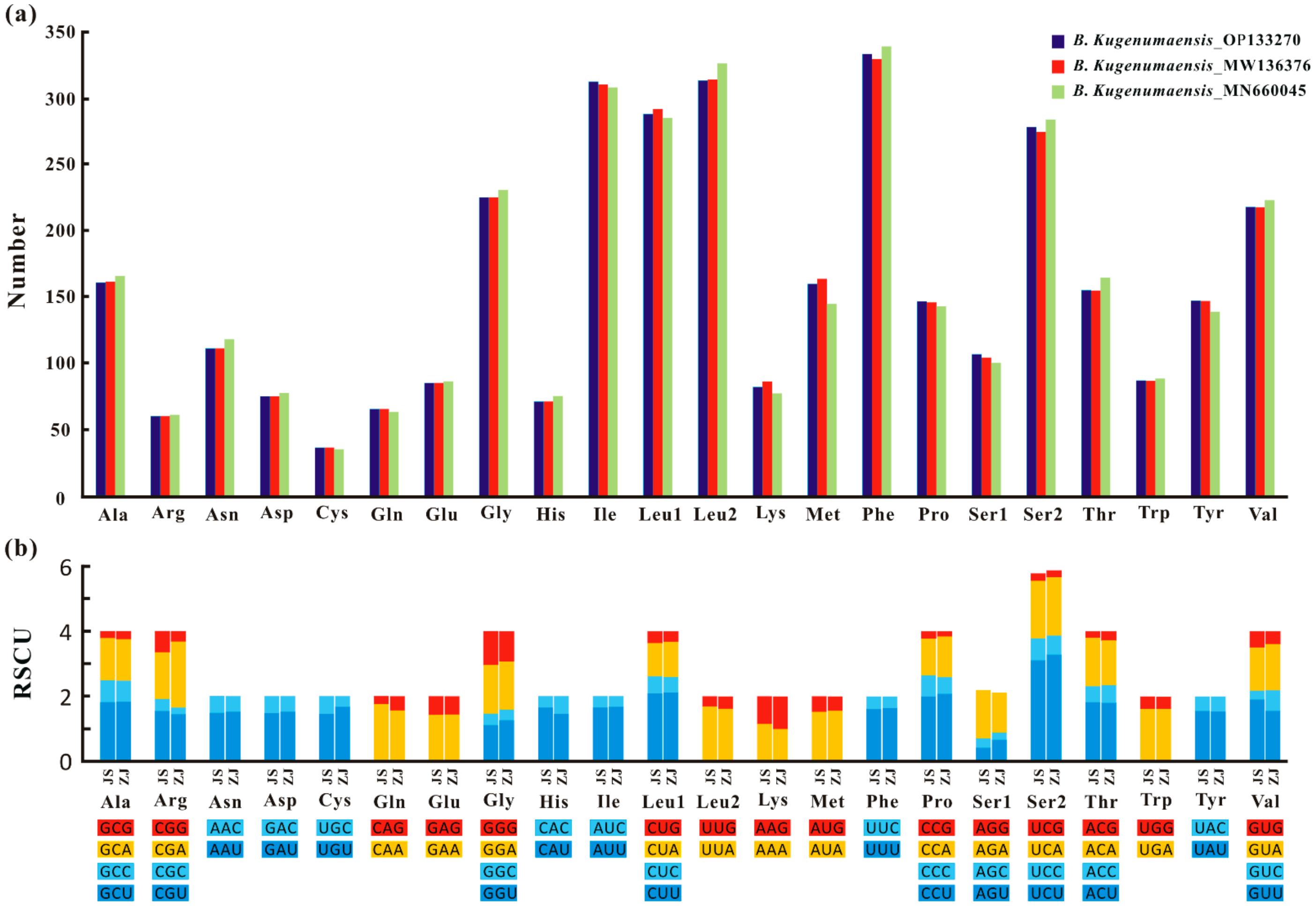

Figure S1. Amino acid composition (a) and relative synonymous codon usage (b) in the mitogenomes of *Branchinella kugenumaensis*

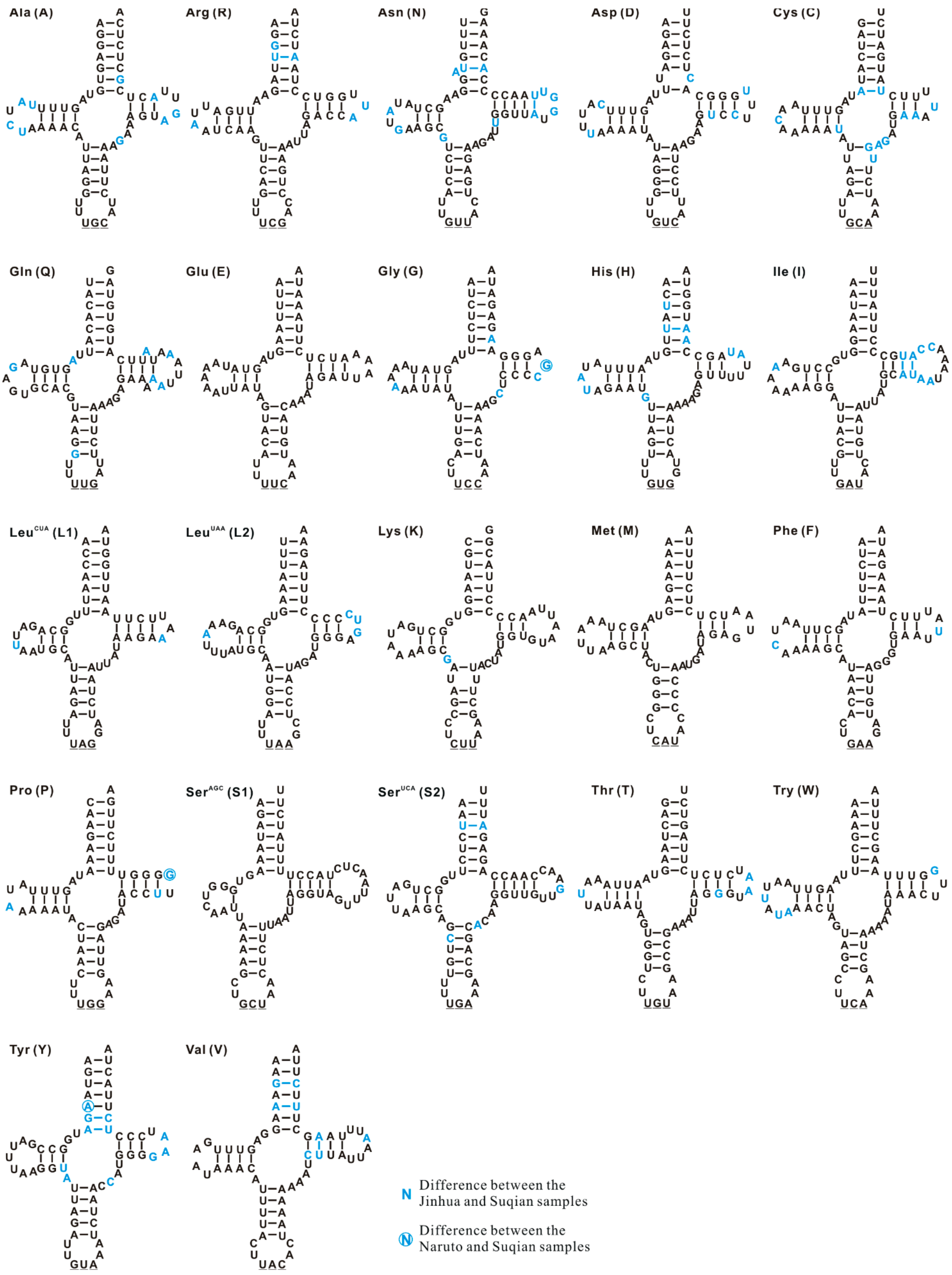

Figure S2. Polymorphisms in inferred secondary structures of 22 transfer RNAs found in the mitogenomes of *B. kugenumaensis*

Table S1. List of primer combinations used to amplify the mitochondrial genome of *B. kugenumaensis*

Fragment	Region	Primer pair	Primer sequence (5′-3′)
BK 1	*cox1*	BK 1F	CCTGATATAGCTTTCCCTCG
		BK 1R	CTTGATTCCTGTAGGAATTGC
BK 2	*cox1-atp6*	BK 2F	CCTGATATAGCTTTCCCTCG
		BK 2R	CTTGATTCCTGTAGGAATTGC
BK 3	*atp6*	BK 3F	CCTGATATAGCTTTCCCTCG
		BK 3R	CTTGATTCCTGTAGGAATTGC
BK 4	*atp6-cox3*	BK 4F	GTTCCTTTAGGAACTCCC
		BK 4R	CTTCTATGGAAAAATGCTCAG
BK 5	*cox3*	BK 5F	GTTCCTTTAGGAACTCCC
		BK 5R	CTTCTATGGAAAAATGCTCAG
BK 6	*cox3-nad5*	BK 6F	GTTCCTTTAGGAACTCCC
		BK 6R	CTTCTATGGAAAAATGCTCAG
BK 7	*nad5*	BK 7F	GTGCATGAGTAAAGAGATG
		BK 7R	CTCTTCTGATGGTTTAAGAC
BK8	*nad5-nad4*	BK 8F	CTACTCGGTTTCTGAGTGCAG
		BK 8R	CTTAAGTTAGGTGGATACGGTC
BK9	*nad4*	BK 9F	GATTTCACGTCAGTCTGTC
		BK 9R	CTTCCCTTATTCCTACTATG
BK10	*nad4-cytb*	BK 10F	GATATTCACTGTTAAATCCAAGG
		BK 10R	GGTTATCAACTGCGAATCCC
BK11	*cytb*	BK 11F	GTATGTACTTCCATGAGGAC
		BK 11R	GACTAATGGGTTAGCTGGTG
BK12	*cytb-16S*	BK 12F	CACCCTTATTTTTCTTTCAAGG
		BK 12R	GTTACCTTAGGGATAACAGC
BK13	*16S*	BK 13F	CTGAACTCAGATCACGTAGG
		BK 13R	GGTCTGGCCTGCCCACTG
BK14	*16S-12S*	BK 14F	AAGAGACAGAAAGTTTCTCG
		BK 14R	CACGGATAGATTTTCTTAG
BK15	*12S*	BK 15F	CTACTTTGTTACGACTTATCTC
		BK 15R	GTGCCAGCATCCGCGGTC
BK16	*12S-cox1*	BK 16F	TAGTGGGGTATCTAATCCCAG
		BK 16R	CTGAGAGTGGGGGATAGACAG

Table S2. COI & 16S rRNA sequences of Anostraca retrieved from GenBank used in this study

Family	Species	COI	16S rRNA
		GenBank ID	GenBank ID
Branchipodidae	*Tanymastix stagnalis* (Linnaeus, 1758)	AY555253	AY555250
Chirocephalidae	*Drepanosurus khankanus* (Takahashi & Moriya in Takahashi et al., 2018)	LC314406	LC314407
	Chirocephalus kerkyrensis Pesta, 1936	JN246503	JN246501
	Chirocephalus marchesonii Ruffo & Vesentini, 1957	JN246530	JN246502
	Artemiopsis stefanssoni (Johansen, 1921)	AF209062	AF209053
	Eubranchipus grubii (Dybowski, 1860)	MT410793	MT410793
	Linderiella occidentalis (Dodds, 1923)		MT010663
	Linderiella sp.	MF458751	
Polyartemiidae	*Polyartemiella hazeni* (Murdoch, 1884)	AF209063	AF209054
Branchinectidae	*Branchinecta lindahli* Packard, 1883	MN992068	MN982244
	Branchinecta mackini Dexter, 1956	HQ966512	MT010652
	Branchinecta paludosa (O. F. Müller, 1788)	MZ853171	MZ853171
Thamnocephalidae	*Thamnocephalus mexicanus* Linder, 1941	KP716860	KP716881
	Thamnocephalus platyurus (Packard, 1877)	KP716880	KP716892
	Phallocryptus fahimii Schwentner, Rudov & Rajaei 2020	MT604043	MT604045
	Phallocryptus tserensodnomi Alonso & Ventura, 2013	KP273592	KP273592
	Branchinella affinis Linder, 1941	AF308959	AF527568
	Branchinella arborea Geddes 1981	AF308962	AF308945
	Branchinella australiensis (Richters, 1876)		AF527557
	Branchinella buchananensis Geddes, 1981	AF308960	AF308943
	Branchinella budjiti Timms, 2001		AF527567
	Branchinella campbelli Timms, 2001		AF527576
	Branchinella frondosa Henry, 1924	AF308958	AF308941
	Branchinella kugenumaensis (Ishikawa, 1895) (Jiangsu, China)	OP133270	OP133270
	Branchinella kugenumaensis (Ishikawa, 1895) (Zhejiang, China)	MN660045	MN660045
	Branchinella kugenumaensis (Ishikawa, 1895) (Japan)	MW136376<br>LC311764	MW136376<br>DQ470607<br>LC311765
	Branchinella longirostris Wolf, 1911	KC335195	AF527575
	Branchinella lyrifera Linder, 1941	AF308957	AF527561
	Branchinella madurai Raj, 1951		JX439912
	Branchinella occidentalis (Dakin, 1914)	AF308964	EF189600
	Branchinella pinnata Geddes, 1981	AF308963	AF527570
	Branchinella proboscida Henry, 1924		AF527573
	Branchinella wellardi Milner, 1929		AF527571
	Branchinella sp. S sensu Pinceel et al., 2012		AF527572
Streptocephalidae	*Streptocephalus cafer* (Lovén, 1847)	MN720104	MN720104
	Streptocephalus dichotomus (Baird, 1860)	JQ340777	KC832403
	Streptocephalus dorothae Mackin, 1942	AF209065	AF209056
	Streptocephalus echinus Bond, 1934		JX439913
	Streptocephalus sirindhornae Sanoamuang, Murugan, Weekers & Dumont, 2000	KP273593	KP273593
	Streptocephalus woottoni Eng, Belk & Eriksen, 1990		KF790574

Table S3. Details of species and mitogenomes of Branchiopoda used in this study

Family	Genus	Species	Length (bp)	Genbanks Accession ID	Reference
Thamnocephalidae	*Branchinella*	*Branchinella kugenumaensis* (Ishikawa, 1895) (Japan)	14123	MW136376	[28]
		Branchinella kugenumaensis (Ishikawa, 1895) (Zhejiang, China)	15127	MN660045	[39]
		Branchinella kugenumaensis (Ishikawa, 1895) (Jiangsu, China)	14126	OP133270	This study
	Phallocryptus	*Phallocryptus tserensodnomi* Alonso & Ventura, 2013	16493	KP273592	[49]
Streptocephalidae	*Streptocephalus*	*Streptocephalus cafer* (Lovén, 1847)	17020	MN720104	[50]
		Streptocephalus sirindhornae Sanoamuang, Murugan, Weekers & Dumont, 2000	16887	NC_026704	[51]
Chirocephalidae	*Drepanosurus*	*Drepanosurus uchidai* (Kikuchi, 1957)	15795	LC633442	[52]
		Drepanosurus hatanakai Takahashi & Hamasaki, in Takahashi et al., 2018	17006	LC633440	
		Drepanosurus asanumai Takahashi, in Takahashi et al., 2018	17503	LC633438	
	Eubranchipus	*Eubranchipus grubii* (Dybowski, 1860)	16328	MT410793	No reference
Branchinectidae	*Branchinecta*	*Branchinecta gaini* Daday, 1910	15536	MZ265218	No reference
		Branchinecta paludosa (Müller 1788)	16059	MZ853171	No reference
Artemiidae	*Artemia*	*Artemia franciscana* Kellogg, 1906	15822	NC_001620	[53]
		Artemia urmiana Gunther, 1899	15945	NC_021382	[54]
		Artemia tibetiana Abatzopoulos, Zhang & Sorgeloos, 1998	15742	NC_021383	[54]
		Artemia sinica Cai, 1989	15689	MK069595	[55]
		Artemia salina (Linneaus, 1758)	15826	MT495441	[56]
		Artemia persimilis Piccinelli & Prosdocimi, 1968	15436	MZ199176	[57]
Limnadiidae	*Limnadia*	*Limnadia lenticularis* (Linnaeus, 1761)	15151	MH618637	[58]
	Gondwanalimnadia	*Gondwanalimnadia* sp.	15661	MN625703.1	[59]
	Leptestheria	*Leptestheria brevirostris* Barnard, 1924	15579	MN548772.1	[60]
Triopsidae	*Triops*	*Triops cancriformis* (Bosc, 1801)	15101	NC_004465	[61]
		Triops australiensis (Spencer & Hall, 1896)	15125	NC_024439	[62]
		Triops longicaudatus (LeConte, 1846)	15060	KM516710	[63]
		Triops longicaudatus (LeConte, 1846)	15058	KM516711	[63]
		Triops longicaudatus (LeConte, 1846)	15115	GU475465	[64]
		Triops granarius (Lucas, 1864)	15121	MF496656	[29]

Table S4. Partition schemes and best-fitting models for phylogenetic analyses

Datasets		Phylogenetic Reconstruction Approaches	
		**Maximum Likelihood Best-fitting model**	**Bayesian Inference Best-fitting model**
Amino acid datasets 27taxa 3,058 sites	*cox1*	mtART+I+G	mtREV+G+I
	cox2	mtREV+G+F	mtREV+G+F
	cox3	mtREV+G+I+F	mtREV+G+I
	cytb	mtREV+G+I+F	mtREV+G+I
	nad1	mtREV+G+I	mtREV+G+I+F
	nad3	mtREV+G+F	mtREV+G+F
	nad4	mtREV+G+I+F	mtREV+G+I+F
	nad5	mtREV+G+I+F	mtREV+G+I+F
	atp6	mtMam+G+I	mtREV+G+I
Nucleotide datasets 27taxa 6,452 sites	The 1st codon of *cox1*	GTR+G+I	SYM+G+I
	The 2nd codon of *cox1*	GTR+G+I	GTR+G+I
	The 1st codon of *cox2*	SYM+G+I	GTR+G+I
	The 2nd codon of *cox2*	TVM+G+I	GTR+G
	The 1st codon of *cox3*	GTR+G+I	GTR+G+I
	The 2nd codon of *cox3*	GTR+G+I	GTR+G+I
	The 1st codon of *cytb*	GTR+G+I	SYM+G+I
	The 2nd codon of *cytb*	GTR+G+I	GTR+G+I
	The 1st codon of *nad1*	GTR+G+I	HKY+G+I
	The 2nd codon of *nad1*	GTR+G	GTR+G+I
	The 2nd codon of *nad2*	TVM+G+I	GTR+G
	The 1st codon of *nad3*	GTR+G+I	HKY+G+I
	The 2nd codon of *nad3*	GTR+G	GTR+G+
	The 1st &2nd codon of *nad4*	GTR+G+I	GTR+G+I
	The 1st &2nd codon of *nad5*	GTR+G	GTR+G+I
	The 1st codon of *atp6*	TVM+G+I	HKY+G+I
	The 2nd codon of *atp6*	GTR+G+I	GTR+G

Table S5. A + T content (%) and skewness levels calculated for major strand of *B. kugenumaensis* and *S. cafer*

Regions	AT%				AT-Skew				GC-Skew			
	BKCJS	BKJT	BKCZJ	SC	BKCJS	BKJT	BKCZJ	SC	BKCJS	BKJT	BKCZJ	SC
Whole genome	67.84	68.17	67.78	68.13	0.048	0.06	0.075	0.038	-0.04	-0.036	-0.047	-0.033
PCGs	67.65	67.67	67.47	67.91	-0.231	-0.232	-0.239	-0.244	-0.058	-0.057	-0.056	-0.043
1st codon position	60.29	60.28	60.15	59.36	-0.126	-0.126	-0.141	-0.125	0.102	0.100	0.107	0.121
2nd codon position	64.59	64.30	63.96	63.86	-0.440	-0.439	-0.436	-0.434	-0.189	-0.182	-0.190	-0.178
3rd codon position	78.06	78.43	78.31	80.50	-0.141	-0.143	-0.152	-0.181	-0.137	-0.135	-0.132	-0.136
rRNA	69.94	69.76	70.09	72.06	-0.093	-0.106	-0.104	-0.097	0.169	0.161	0.204	0.191
tRNA	68.63	68.80	68.27	67.65	0.019	0.020	0.010	0.026	0.158	0.127	0.113	0.115
atp6	66.67	66.97	67.12	66.67	-0.167	-0.150	-0.116	-0.183	-0.241	-0.226	-0.204	-0.178
atp8	73.34	73.72	72.44	77.56	-0.216	-0.165	-0.080	-0.058	-0.231	-0.171	-0.116	-0.314
cox1	63.46	63.73	62.88	64.19	-0.161	-0.161	-0.164	-0.203	-0.002	-0.001	-0.002	-0.002
cox2	64.33	64.05	66.22	66.52	-0.141	-0.146	-0.091	-0.108	-0.057	-0.041	-0.052	-0.044
cox3	65.26	65.13	64.37	65.77	-0.190	-0.196	-0.194	-0.192	0.029	0.040	0.011	-0.022
cytb	63.23	63.40	63.40	64.90	-0.169	-0.168	-0.160	-0.153	-0.132	-0.123	-0.123	-0.134
nad1	67.92	67.79	68.12	66.78	-0.333	-0.343	-0.350	-0.360	0.021	0.035	0.046	0.044
nad2	70.75	70.96	72.20	72.32	-0.202	-0.213	-0.218	-0.238	-0.022	-0.043	-0.041	-0.029
nad3	73.98	74.27	72.73	71.35	-0.138	-0.134	-0.145	-0.148	-0.034	-0.045	0.075	0.041
nad4	70.27	70.35	70.85	70.67	-0.361	-0.364	-0.388	-0.379	-0.127	-0.153	-0.132	-0.116
nad4L	72.03	71.65	72.16	74.31	-0.404	-0.412	-0.413	-0.464	-0.123	-0.135	-0.127	0.164
nad5	68.82	68.78	67.41	68.02	-0.293	-0.296	-0.338	-0.303	0.008	0.002	-0.017	0.033
nad6	76.29	75.62	73.6	72.26	-0.120	-0.136	-0.210	-0.201	-0.170	-0.174	-0.237	-0.226

Taxa labels: BKCJS = *B. kugenumaensis* (Suqian, Jiangsu, China), BKJT = *B. kugenumaensis* (Naruto, Tokushima Prefecture, Japan), BKCZJ = *B. kugenumaensis* (Jinhua, Zhejiang, China), SC = *S. cafer*.

Table S6. Nucleotide identity (%) and number of codon substitutions for each PCG of three *B. kugenumaensis* mtgenomes

Genes	MN660045 vs MW136376			OP133270 vs MW136376		
	Identity (%)	No. synonymous codon substitutions	No. nonsynonymous codon substitutions	Identity (%)	No. synonymous codon substitutions	No. nonsynonymous codon substitutions
atp6	76.97	95	28	99.55	3	0
atp8	66.04	6	13	99.37	0	1
cox1	83.64	221	9	99.35	8	2
cox2	82.39	100	5	99.13	4	2
cox3	83.29	97	9	99.36	5	0
cob	77.92	182	26	99.12	5	4
nad1	78.71	109	36	99.11	5	2
nad2	73.99	104	50	98.87	6	4
nad3	78.55	40	13	99.70	0	1
nad4	74.73	146	64	98.58	13	2
nad4L	74.24	24	12	98.11	2	3
nad5	74.82	190	99	98.63	12	8
nad6	70.22	48	36	98.21	3	4
